# Supplementary material for: Health-state utility of patients with HER2-positive breast cancer in Vietnam: A multicenter cross-sectional study
Source: PLoS One. 2024 May 14;19(5):e0303011. doi: 10.1371/journal.pone.0303011 (PMC11093329; doi:10.1371/journal.pone.0303011)
Supplement: Attachment — Submitted filename: PONE-D-23-42055.pdf [file pone.0303011.s002.pdf]

PLOS ONE

Health-state utility of patients with HER2-positive breast cancer in Vietnam: a multicenter cross-sectional study

--Manuscript Draft--

Manuscript Number: PONE-D-23-42055

Article Type: Research Article

Full Title: Health-state utility of patients with HER2-positive breast cancer in Vietnam: a multicenter cross-sectional study

Short Title: HER2-positive breast cancer patients' health-state utility

Corresponding Author: Dai Xuan Dinh, MS
Hanoi University of Pharmacy
Hanoi, Hanoi VIET NAM

Keywords: HER2-positive, breast cancer, quality of life, systemic treatment, utility, Vietnam

Abstract: Abstract

Objective
To investigate the health-state utility and its associated factors among Vietnamese patients with human epidermal growth factor receptor 2 (HER2)-positive breast cancer.
Methods
We ~~directly~~ interviewed 301 HER2-positive breast cancer patients recruited from one national cancer hospital in the north and one oncology center of a tertiary referral hospital in the central part of Vietnam. The patient's health-state utility was measured via the EQ-5D-5L instrument. The Mann-Whitney U and Kruskal-Wallis tests were employed to compare the differences in utility scores between two groups and among three groups or more, respectively. Factors associated with patients' heath-state utility were identified via Tobit regression models.
Results
Pain/discomfort (56.1%) and anxiety/depression (39.5%) were the two issues that patients suffered from the most, especially among metastatic breast cancer patients. The severity of distress (depression, anxiety, and stress) in patients was relatively mild. Of 301 patients, their average utility score was 0.86±0.17 (range: 0.03-1.00), and the average EQ-visual analogue scale (VAS) score was 69.12±12.60 (range: 30-100). These figures were 0.79±0.21 and 65.15±13.20 for 102 metastatic breast cancer patients, significantly lower than those of 199 non-metastatic cancer patients (0.89±0.13 and 71.13±11.78) ($p<0.001$), respectively. As per the multivariate Tobit regression model, lower health-state utility scores were significantly associated with older age ($p=0.003$), lower education level ($p=0.004$), lower monthly income ($p=0.027$), metastatic cancer ($p<0.001$), lower EQ-VAS score ($p<0.001$), and more severe level of distress ($p<0.001$).
Conclusions
This research provides some essential information about the health-state utility and its associated factors among metastatic and non-metastatic HER2-positive breast cancer patients. These data are fundamental for further health technology assessment and healthcare programs for this patient group in Vietnam.

Order of Authors:
Tram Nguyen Nguyet Luu
Dai Xuan Dinh, MS
Thinh Xuan Tran
Thang Binh Tran
Huong Thanh Tran
Kiet Huy Tuan Pham
Huong Thi Thanh Nguyen

Opposed Reviewers:

Powered by Editorial Manager® and ProduXion Manager® from Aries Systems Corporation

Additional Information:	
**Question**	**Response**
**Financial Disclosure**<br><br>Enter a financial disclosure statement that describes the sources of funding for the work included in this submission. Review the submission guidelines for detailed requirements. View published research articles from *PLOS ONE* for specific examples.<br><br>This statement is required for submission and **will appear in the published article** if the submission is accepted. Please make sure it is accurate.<br><br>**Funded studies**<br>Enter a statement with the following details:<br>• Initials of the authors who received each award<br>• Grant numbers awarded to each author<br>• The full name of each funder<br>• URL of each funder website<br>• Did the sponsors or funders play any role in the study design, data collection and analysis, decision to publish, or preparation of the manuscript?<br><br>Did you receive funding for this work?	The author(s) received no specific funding for this work.
**Competing Interests**<br><br>Use the instructions below to enter a competing interest statement for this submission. On behalf of all authors, disclose any competing interests that could be perceived to bias this work—acknowledging all financial support and any other relevant financial or non-financial competing interests.<br><br>This statement is required for submission and **will appear in the published article** if the submission is accepted. Please make sure it is accurate and that any funding sources listed in your Funding Information	The authors declare that they have no competing interests.

later in the submission form are also declared in your Financial Disclosure statement.<br><br>View published research articles from *PLOS ONE* for specific examples.<br><br>**NO authors have competing interests**<br><br>Enter: *The authors have declared that no competing interests exist.*<br><br>**Authors with competing interests**<br><br>Enter competing interest details beginning with this statement:<br><br>*I have read the journal's policy and the authors of this manuscript have the following competing interests: [insert competing interests here]*<br><br>* typeset	
**Ethics Statement**<br><br>Enter an ethics statement for this submission. This statement is required if the study involved:<br><br>• Human participants<br>• Human specimens or tissue<br>• Vertebrate animals or cephalopods<br>• Vertebrate embryos or tissues<br>• Field research<br><br>Write "N/A" if the submission does not require an ethics statement.<br><br>General guidance is provided below. Consult the submission guidelines for detailed instructions. **Make sure that all information entered here is included in the Methods section of the manuscript.**	This research was part of a project (named: Cost-effectiveness analysis of some breast cancer treatment regimens in Vietnam) approved by the ethics committee of the Hue University of Medicine and Pharmacy, Hue University, Vietnam (reference number: H2022/495, dated 20th September 2022). The authors also received the permission of the board of directors of two hospitals where data were collected. The participants (patients with HER2-positive breast cancer) gave written informed consent before participating in this research. The authors had access to medical records and information that could identify individual participants during the data collection. However, patients' personal information will be kept confidential. The time for patient recruitment and access to medical records was from November 2022 to April 2023.

Powered by Editorial Manager® and ProduXion Manager® from Aries Systems Corporation

**Format for specific study types**<br><br>**Human Subject Research (involving human participants and/or tissue)**<br>• Give the name of the institutional review board or ethics committee that approved the study<br>• Include the approval number and/or a statement indicating approval of this research<br>• Indicate the form of consent obtained (written/oral) or the reason that consent was not obtained (e.g. the data were analyzed anonymously)<br><br>**Animal Research (involving vertebrate animals, embryos or tissues)**<br>• Provide the name of the Institutional Animal Care and Use Committee (IACUC) or other relevant ethics board that reviewed the study protocol, and indicate whether they approved this research or granted a formal waiver of ethical approval<br>• Include an approval number if one was obtained<br>• If the study involved *non-human primates*, add *additional details* about animal welfare and steps taken to ameliorate suffering<br>• If anesthesia, euthanasia, or any kind of animal sacrifice is part of the study, include briefly which substances and/or methods were applied<br><br>**Field Research**<br><br>Include the following details if this study involves the collection of plant, animal, or other materials from a natural setting:<br>• Field permit number<br>• Name of the institution or relevant body that granted permission	
**Data Availability**<br><br>Authors are required to make all data underlying the findings described fully available, without restriction, and from the time of publication. PLOS allows rare exceptions to address legal and ethical concerns. See the PLOS Data Policy and FAQ for detailed information.	No - some restrictions will apply

Powered by Editorial Manager® and ProduXion Manager® from Aries Systems Corporation

A Data Availability Statement describing where the data can be found is required at submission. Your answers to this question constitute the Data Availability Statement and **will be published in the article**, if accepted.<br><br>**Important:** Stating 'data available on request from the author' is not sufficient. If your data are only available upon request, select 'No' for the first question and explain your exceptional situation in the text box.<br><br>Do the authors confirm that all data underlying the findings described in their manuscript are fully available without restriction?	
**Describe where the data may be found in full sentences. If you are copying our sample text, replace any instances of XXX with the appropriate details.**<br><br>• If the data are **held or will be held in a public repository**, include URLs, accession numbers or DOIs. If this information will only be available after acceptance, indicate this by ticking the box below. For example: *All XXX files are available from the XXX database (accession number(s) XXX, XXX.*).<br>• If the data are all contained **within the manuscript and/or Supporting Information files**, enter the following: *All relevant data are within the manuscript and its Supporting Information files.*<br>• If neither of these applies but you are able to provide **details of access elsewhere**, with or without limitations, please do so. For example:<br><br>*Data cannot be shared publicly because of [XXX]. Data are available from the XXX Institutional Data Access / Ethics Committee (contact via XXX) for researchers who meet the criteria for access to confidential data.*<br><br>*The data underlying the results presented in the study are available from (include the name of the third party*	The dataset underpinning findings in this manuscript contain potentially identifying and sensitive patient information. However, the datasets used and/or analyzed during the current study are available from the corresponding author upon reasonable request.

and contact information or URL).<br>• This text is appropriate if the data are owned by a third party and authors do not have permission to share the data.<br><br>* typeset	
Additional data availability information:	

Powered by Editorial Manager® and ProduXion Manager® from Aries Systems Corporation

HANOI UNIVERSITY OF PHARMACY
Address: 13-15 Le Thanh Tong, Hoankiem, Hanoi, Vietnam
Website: www.hup.edu.vn Tel: 844 3933 1012 Fax: 844 3933 2332

Dec 14, 2023

Dear Editors of **PLOS ONE**.

We are pleased to submit our manuscript “**Health-state utility of patients with HER2-positive breast cancer in Vietnam: a multicenter cross-sectional study**” for publication in **PLOS ONE** as an original research article.

In 2020, as per GLOBOCAN, breast cancer was the most commonly diagnosed cancer and ranked fifth in the leading causes of cancer deaths. HER2-positive breast cancer accounts for 15-20%, is more likely to spread from the breast to other areas of the body (metastasis), and recurs than other types of breast cancers. Patients with HER2-positive breast cancer may have poorer prognoses and shorter overall and disease-free survival. In this study, we measured the health-state utility and its associated factors among Vietnamese patients with HER2-positive breast cancer. We used the EQ-5D-5L and DASS-10 instruments to directly interview 301 patients recruited from one national cancer hospital in the north and one oncology center of a tertiary referral hospital in the central part of Vietnam.

Our findings showed that pain/discomfort (56.1%) and anxiety/depression (39.5%) were the two issues that patients suffered from the most, especially among metastatic breast cancer patients. The severity of distress (depression, anxiety, and stress) in patients was relatively mild. Of 301 patients, their average utility score was 0.86±0.17 (range: 0.03-1.00), and the average EQ-visual analogue scale (VAS) score was 69.12±12.60 (range: 30-100). These figures were 0.79±0.21 and 65.15±13.20 for 102 metastatic breast cancer patients, significantly lower than those of 199 non-metastatic cancer patients (0.89±0.13 and 71.13±11.78) ($p<0.001$), respectively. As per the multivariate Tobit regression model, lower health-state utility scores were significantly associated with older age ($p=0.003$), lower education level ($p=0.004$), lower monthly income ($p=0.027$), metastatic cancer ($p<0.001$), lower EQ-VAS score ($p<0.001$), and more severe level of distress ($p<0.001$). In conclusion, our research provides some essential information about the health-state utility and its associated factors among metastatic and non-metastatic HER2-positive breast cancer patients. These data are fundamental for further health technology assessment and healthcare programs for this patient group in Vietnam.

We strongly believe that **PLOS ONE** would be an outstanding forum to disseminate our findings to readers, especially medical personnel and policymakers. We confirm that this manuscript has not been published elsewhere and has not been under consideration by another journal. We declare that we have no conflicts of interest in this work. All authors attest they meet the ICMJE criteria for authorship. All authors have seen and approved the final version of the paper and have agreed to its submission for publication in **PLOS ONE**.

Thank you for considering our manuscript.

Best regards.

Dai Xuan Dinh, Hanoi University of Pharmacy, Vietnam.
Email: daidinh.hup@gmail.com / daidx@hup.edu.vn Phone: +84 39 456 1025

Manuscript Click here to access/download;Manuscript;Manuscript.docx

Title page

Health-state utility of patients with HER2-positive breast cancer in Vietnam: a multicenter cross-sectional study

Short title: HER2-positive breast cancer patients' health-state utility

Tram Nguyen Nguyet Luu[1], Dai Xuan Dinh[2*], Thinh Xuan Tran[3], Thang Binh Tran[4], Huong Thanh Tran[5,6], Kiet Huy Tuan Pham[7], Huong Thi Thanh Nguyen[2]**

[1] Faculty of Pharmacy, Hue University of Medicine and Pharmacy, Hue University, Hue, Vietnam.

[2] Faculty of Pharmaceutical Management and Economics, Hanoi University of Pharmacy, Hanoi, Vietnam

[3] Department of Anesthesiology and Intensive Care, Hue University of Medicine and Pharmacy, Hue University, Hue, Vietnam.

[4] Faculty of Public Health, Hue University of Medicine and Pharmacy, Hue University, Hue, Vietnam.

[5] Vietnam National Cancer Institute, Vietnam National Cancer Hospital, Hanoi, Vietnam

[6] Department of Medical Ethics and Medical Sociology, Hanoi Medical University, Hanoi, Vietnam

[7] Department of Health Economics, Hanoi Medical University, Viet Nam.

Corresponding authors:

*: daidinh.hup@gmail.com

**: huongnguyenthanh@hup.edu.vn

Abstract

Objective

To investigate the health-state utility and its associated factors among Vietnamese patients with human epidermal growth factor receptor 2 (HER2)-positive breast cancer.

Methods

We directly interviewed 301 HER2-positive breast cancer patients recruited from one national cancer hospital in the north and one oncology center of a tertiary referral hospital in the central part of Vietnam. The patient's health-state utility was measured via the EQ-5D-5L instrument. The Mann-Whitney U and Kruskal-Wallis tests were employed to compare the differences in utility scores between two groups and among three groups or more, respectively. Factors associated with patients' heath-state utility were identified via Tobit regression models.

Results

Pain/discomfort (56.1%) and anxiety/depression (39.5%) were the two issues that patients suffered from the most, especially among metastatic breast cancer patients. The severity of distress (depression, anxiety, and stress) in patients was relatively mild. Of 301 patients, their average utility score was 0.86±0.17 (range: 0.03-1.00), and the average EQ-visual analogue scale (VAS) score was 69.12±12.60 (range: 30-100). These figures were 0.79±0.21 and 65.15±13.20 for 102 metastatic breast cancer patients, significantly lower than those of 199 non-metastatic cancer patients (0.89±0.13 and 71.13±11.78) (p<0.001), respectively. As per the multivariate Tobit regression model, lower health-state utility scores were significantly associated with older age (p=0.003), lower education level (p=0.004), lower monthly income (p=0.027), metastatic cancer (p<0.001), lower EQ-VAS score (p<0.001), and more severe level of distress (p<0.001).

Conclusions

This research provides some essential information about the health-state utility and its associated factors among metastatic and non-metastatic HER2-positive breast cancer patients. These data are fundamental for further health technology assessment and healthcare programs for this patient group in Vietnam.

Keywords: HER2-positive, breast cancer, quality of life, systemic treatment, utility, Vietnam.

Introduction

Globally, it is estimated that there were approximately 2.3 million new cases and nearly 685 thousand deaths by reason of breast cancer in 2020. This disease was the most commonly diagnosed cancer (both sexes, all ages: 11.7% of all cancer cases; women: 24.5%) and ranked fifth in the leading causes of cancer deaths. The incidence and mortality rates for this breast disease were 47.8 and 13.6 per 100,000, respectively [1]. In Vietnam, about 21,555 new cases and 9,345 deaths involving breast cancer were reported in 2020. This breast disease ranked third in the new cancer cases and fourth in the number of cancer deaths (incidence rate: 34.2 per 100,000; mortality rate: 13.8 per 100,000) [2]. ~~The most substantial risk factor for breast cancer is female gender. This cancer disease can occur among women at any age after puberty in every country around the world [3].~~

The HER2 (human epidermal growth factor receptor 2) receptor, which is a transmembrane glycoprotein with tyrosine kinase activity, plays a role in controlling epithelial cell proliferation and differentiation [4]. The HER2 gene is amplified from two-fold to more than 20-fold among roughly 15-20% of breast cancer patients. The amplification of this gene gives rise to protein expression and the increasing number of receptors at the cell surface, thereby contributing to excessive cellular division and tumor formation [5, 6]. HER2-positive breast cancer is more likely to spread from the breast to other areas of the body (metastasis) and recurs than other types of breast cancers. Patients with HER2-positive breast cancer may have poorer prognoses and shorter overall and disease-free survival [7, 8]. Luckily, the discovery of the HER2 gene and the HER2-targeted drugs ushered in a new chapter for patients with both early-stage and metastatic HER2-positive breast cancer. These drugs are recommended for use and feature in numerous countries' standard treatment regimens. Using HER2-targeted drugs (such as trastuzumab, pertuzumab, lapatinib, and neratinib) can make a contribution to improving the treatment effectiveness, reducing the risks of recurrence and mortality, and prolonging survival for HER2-positive breast cancer patients [9-11].

Besides the targets mentioned above, health-related quality of life is also a vital outcome employed to assess the effectiveness and efficacy of cancer treatment therapies. There are a plethora of previous studies conducted to assess the quality of life and health-state utility of breast cancer patients, including several studies in Vietnam [12-14]. However, most of them focused on the general population of breast cancer patients. The number of studies for HER2-positive breast cancer patients is negligible. In addition, the treatment costs of HER2-targeted drugs are exorbitant. As a result, these medications are of great interest to researchers and

policymakers. Measuring the health-related quality of life and health-state utility of HER2-positive breast cancer patients is essential for the health technology assessment of anti-HER2 agents. These data can be used to compute significant indicators and parameters, thereby providing reliable evidence supporting the decision-making related to health insurance coverage policies. This research was conducted to measure the health-state utility and its associated factors among Vietnamese patients with HER2-positive breast cancer.

Methods

Ethical Approval

This research was part of a project (~~named: Cost-effectiveness analysis of some breast cancer treatment regimens in Vietnam~~) approved by the ethics committee of the Hue University of Medicine and Pharmacy, Hue University, Vietnam (reference number: H2022/495, dated 20th September 2022). The authors also received the permission of the board of directors of two hospitals where data were collected. The participants (patients with HER2-positive breast cancer) gave written informed consent before participating in this research. The authors had access to medical records and information that could identify individual participants during the data collection. However, patients' personal information will be kept confidential. The time for patient recruitment and access to medical records was from November 2022 to April 2023.

Study design and setting

This cross-sectional study was conducted in two hospitals to collect data on patients with HER2-positive breast cancer. The first hospital was the *Vietnam National Cancer Hospital* in northern Vietnam. This is Vietnam's biggest specialized oncology hospital, with approximately 2,000 inpatient beds. The second health facility was the *Oncology Center* of *Hue Central Hospital*, a tertiary referral hospital with about 4,500 inpatient beds in the central region of Vietnam. This center has about 500 standard hospital beds and is one of Vietnam's leading cancer treatment facilities.

Participants and the sample size

The study population was patients with HER2-positive breast cancer, including early (I, II, and III) and late stages (IV). Two standard tests employed to identify whether or not breast cancer is HER2-positive were the IHC test (ImmunoHisstoChemistry) and (2) the FISH test (Fluorescence In Situ Hybridization). The latter is more accurate but expensive and takes longer to have results than the former. Patient inclusion criteria included (1)

inpatients aged 18 or older, (2) being diagnosed with HER2-positive breast cancer (having an IHC result of 3+ or an IHC result of 2+ along with a FISH test score of positive), (3) receiving systemic therapy for adjuvant or metastatic treatment (including chemotherapy only, chemotherapy in combination with HER2-targeted therapy, or HER2-targeted therapy with/without hormone therapy), and (4) having at least three cycles of treatment before the time of interviewing. Patients were excluded if they did not concur to participate in this research, did not give written informed consent, and could not finish the questionnaire by virtue of feeling haggard or having psychological issues.

The sample size was computed via the following formula: $n = Z^2_{(1-\alpha/2)}.\sigma^2/(\varepsilon^2.\mu^2)$, including n: the smallest number of participants, $Z_{(1-\alpha/2)}$ = 1,96 (α = 0.05), σ (standard deviation) = 0.22 (from a previous study [14]), ε (margin of error) = 5%, and μ = 0,74 [14], the minimum sample size was 136 patients. In November 2022, after an initial screening, there were approximately 446 eligible inpatients in two selected hospitals. We strived to approach nearly all of them[illegible]owever, only 301 patients concurred to participate in this research (response rate = 67.5%). Data collectors (the authors) introduced the study objectives and procedures to patients with HER2-positive breast cancer. Then, they were invited to participate in this research. Those agreeing to take part in this research gave us their written informed consent and then got a face-to-face interview with data collectors. After that, data collectors collected additional data from inpatients' medical records.

The questionnaire and measurements

Patient's demographic characteristics were mainly collected via a direct interview (including year of birth, residence, education level, job/occupation, marital status, whether or not giving birth, patient monthly income, and household monthly income). Patient's clinical characteristics and their treatment process were gathered from medical records, including the stage of cancer (metastatic or non-metastatic), type of metastasis (relapsed or de novo), site of metastasis, comorbidity, menopausal status, hormone receptor (positive or negative), and treatment regimens. Many previous studies showed that breast cancer patients suffered from mental health and psychological issues [15-17]. In this study, the DASS-10 (Depression Anxiety Stress Scale) was utilized to measure patients' level of distress. The validity and reliability of DASS-10 was demonstrated in a previous study [18]. We received the DASS-10 developers' permission to translate and use this scale. The linguistic validation of the DASS-10 consisted of three steps: forward translation, backward translation, and testing. This questionnaire consists of ten questions divided into two subscales (including depression and anxiety-stress). For each

question, patients had four answering options, including “never”, “sometimes”, “often”, and “almost always”. The total score of a patient can range from 0 to 30 (reflecting the overall distress: (1) mild/subclinical: 6 or less, (2) moderate: 7 – 12, and (3) severe: 13 or higher) [19].

The primary outcome was patients’ health-state utility measured via the EQ-5D-5L questionnaire - one of the multi-attribute utility instruments recommended for use in national health technology guidelines [20]. This instrument comprises two parts: (1) the EQ visual analogue scale (VAS) and (2) the EQ-5D descriptive system. The former is a vertical visual analog scale ranging from 0 (The worst health a patient can imagine) to 100 (The best health a patient can imagine). This scale reflects the patient’s health based on their own judgment (self-rated health). The latter includes five dimensions: (1) mobility, (2) self-care, (3) usual activities, (4) pain/discomfort, and (5) anxiety/depression. One dimension consists of five levels of problems: (1) no, (2) slight, (3) moderate, (4) severe, and (5) extreme/unable. Patients were also categorized into two groups for each dimension: no problems if they chose the “no” option and having problems if they chose one of the four remaining options. The Vietnamese version of EQ-5D-5L and the value set for the general population in Vietnam (scoring) are available [21, 22]. The use of this questionnaire was approved by the EuroQoL Research Foundation (request registration number 50956, dated 25 Jul 2022).

Data analysis

After being collected, data were entered into an Excel file and analyzed with SPSS version 26 and STATA version 15. Mean (standard deviation) or median (25th-75th) was used to report numeric variables (such as patient’s age), while categorical variables (such as occupation) were described via numbers and percentages. The relationship between two categorical variables was identified using a Chi-squared or Fisher’s exact test. Regarding numeric variables, the Kolmogorov-Smirnov test was used to check the normal distribution of data. By reason of the non-normal distribution, the Mann-Whitney U and Kruskal-Wallis rank-sum tests were employed to compare the differences in utility scores between two groups and among three groups or more, respectively. In addition, factors associated with patients’ health-state utility were identified using Tobit regression models that describe the relationship between a truncated or censored continuous variable (a dependent variable) and independent variables, in line with several previous studies [14, 23]. A p-value<0.05 was regarded as statistical significance.

Results

The general characteristics of participants

Of 301 participants, there were 199 non-metastatic breast cancer patients (66.1%) and 102 metastatic breast cancer patients (33.9%). On average, patients' age was 51.1±9.5 years old. Roughly 88.0% of patients aged 40 or older. About two-thirds came from rural areas (61.5%) and had an education level of secondary school, high school, or college (71.1%). Most patients got married (95.3%) and gave birth (99.0%). Common occupations were farmers (28.9%), employees/business people (25.2%), and freelancers (24.9%). A quarter had a monthly income of less than two million Vietnam dongs (24.9%), while this figure of nearly two-fifths was higher than three million Vietnam dongs (57.5%). Patients' average income per month was approximately 5.0±4.7 million Vietnam dongs. Between the two patient groups (non-metastatic and metastatic), there were significant differences in education level (p=0.021), occupation (p<0.001), giving birth (=0.038), and patient's income per month (p=0.009) (Table 1).

Table 1. Demographic characteristics of participants (n=301 patients)

**Demographic characteristics**		**Patients n (%)**			**p-value**
		**All**	**Non-metastatic**	**Metastatic**	
**Age**	<40	36 (12.0)	24 (12.1)	12 (11.8)	0.070
	40-49	88 (29.2)	66 (33.2)	22 (21.6)	
	50-59	116 (38.5)	76 (38.2)	40 (39.2)	
	≥60	61 (20.3)	33 (16.6)	28 (27.5)	
**Residence**	Urban	116 (38.5)	75 (37.7)	41 (40.2)	0.672
	Rural	185 (61.5)	124 (62.3)	61 (59.8)	
**Education level**	Primary school or lower	15 (5.0)	8 (4.1)	7 (6.9)	**0.021**
	Secondary school	102 (33.9)	59 (29.6)	43 (42.2)	
	High school or college	139 (37.2)	95 (47.7)	44 (43.1)	
	University or higher	45 (15.0)	37 (18.6)	8 (7.8)	

Demographic characteristics		Patients n (%)			p-value
		All	Non-metastatic	Metastatic	
**Job (occupation)**	Employment or business	76 (25.2)	64 (32.2)	12 (11.8)	**<0.001**
	Agriculture	87 (28.9)	44 (22.1)	43 (42.2)	
	Housework	22 (7.3)	13 (6.5)	9 (8.8)	
	Freelancers	75 (24.9)	51 (25.6)	24 (23.5)	
	Retired	41 (13.6)	27 (13.6)	14 (13.7)	
**Marital status**	Unmarried or divorced	14 (4.7)	7 (3.5)	7 (6.9)	0.247
	Married	287 (95.3)	192 (96.5)	95 (93.1)	
**Gave birth**	No	3 (1.0)	0 (0.0)	3 (2.9)	**0.038**
	Yes	298 (99.0)	199 (100.0)	99 (97.1)	
**Patient monthly income (mVND)***	No income	49 (16.3)	28 (14.1)	21 (20.6)	**0.009**
	≤2	26 (8.6)	15 (7.5)	11 (10.8)	
	>2 to 3	48 (15.9)	25 (12.6)	23 (22.5)	
	>3	173 (57.5)	128 (64.3)	45 (44.1)	
	No answering	5 (1.7)	3 (1.5)	2 (2.0)	
**Household monthly income (mVND)***	≤4	31 (10.3)	17 (8.5)	14 (13.7)	0.202
	>4 to 6	25 (8.3)	15 (7.5)	10 (9.8)	
	>6	194 (64.5)	135 (67.8)	59 (57.9)	
	No answering	51 (16.9)	32 (16.1)	19 (18.6)	
*1 mVND (million Vietnam dongs) = 41.27 US dollars.					

Of 102 metastatic breast cancer patients, nearly a third had de novo metastatic cancer. More than three-quarters had visceral and/or central nervous system metastasis. Among all patients, 31.2% had at least one

comorbidity, and 57.8% were menopausal women. The percentage of patients with hormone receptor-positive breast cancer (49.2%) was relatively equal to that of patients with hormone receptor-negative breast cancer (50.8%). Regarding the treatment regimens, nearly half of non-metastatic breast cancer patients (47.2%) were given HER2-targeted therapy only or in combination with hormone therapy. Meanwhile, a majority of metastatic breast cancer patients received chemotherapy only (53.9%) or in combination with HER2-target therapy (32.4%). Between the two patient groups (non-metastatic and metastatic), there were significant differences in comorbidity (p=0.039), menopausal status (p=0.048), hormone receptor (p=0.010), and received treatment regimens (p<0.001) (Table 2).

Table 2. Clinical characteristics of participants (n=301 patients)

**Demographic characteristics**		**Patients n (%)**			**p-value**
		**All**	**Non-metastatic**	**Metastatic**	
**Stage of cancer (tumor stage)**	I, II, or III	199 (69.1)	199 (100.0)	-	
	IV	102 (33.9)	-	102 (100.0)	
**Type of metastasis**	Relapsed	-	-	69 (67.6)	
	De novo	-	-	33 (32.4)	
**Site of metastasis**	Visceral or central nervous system	-	-	78 (76.5)	
	Non-visceral	-	-	24 (23.5)	
**Comorbidity**	Yes	94 (31.2)	70 (35.2)	24 (23.5)	**0.039**
	No	207 (68.8)	129 (64.8)	78 (76.5)	
**Menopause**	Yes	174 (57.8)	107 (53.8)	67 (65.7)	**0.048**
	No	127 (42.2)	92 (46.2)	35 (34.3)	
**Hormone receptor**	Positive	148 (49.2)	111 (55.8)	37 (36.3)	**0.010**
	Negative	153 (50.8)	88 (44.2)	65 (63.7)	
**Treatment**	Chemotherapy only	115 (38.2)	60 (30.2)	55 (53.9)	**<0.001**

Demographic characteristics		Patients n (%)			p-value
		All	Non-metastatic	Metastatic	
**regimen**	Chemotherapy + HER2-targeted therapy	78 (25.9)	45 (22.6)	33 (32.4)	
	HER2-targeted therapy with or without hormone therapy	108 (35.9)	94 (47.2)	14 (13.7)	
HER2: human epidermal growth factor receptor 2.					

Patients' health profile across five health dimensions of EQ 5D-5L and DASS-10

Among all 301 patients, pain/discomfort (56.1%) and anxiety/depression (39.5%) were the two issues that many patients suffered from most. In comparison with the non-metastatic group, the percentages of patients with metastatic breast cancer having problems involving usual activities, pain/discomfort, and anxiety/depression were significantly higher (p=0.012, p<0.001, and p<0.001, respectively). Regarding these three dimensions, the number of metastatic breast cancer patients having severe or extreme problems was also higher than that of the non-metastatic patient group (usual activities: 5.9% and 0.0%; pain/discomfort: 9.8% and 4.0%; and anxiety/depression: 7.9% and 4.0%, respectively). Among all 301 patients with HER2-positive breast cancer, their average utility score was 0.86±0.17 (range: 0.03-1.00), and the average EQ-VAS score was 69.12±12.60 (range: 30-100). These scores of 102 metastatic cancer patients (0.79±0.21 and 65.15±13.20) were significantly lower than those of 199 non-metastatic cancer patients (0.89±0.13 and 71.13±11.78) (p<0.001 and p<0.001), respectively. In addition, the average DASS-10 score of the former was slightly higher than that of the latter, but this difference was insignificant (p=0.245) (Table 3, Figure 1).

Table 3. Patients' health profile across five health dimensions of EQ 5D-5L and DASS-10

Dimensions		Patients n (%)			p-value
		All	Non-metastatic	Metastatic	
**Morbidity**	No problems	233 (77.4)	160 (80.4)	73 (71.6)	0.083
	Having problems	68 (22.6)	39 (19.6)	29 (28.4)	

Dimensions		Patients n (%)			p-value
		All	Non-metastatic	Metastatic	
**Self-care**	No problems	265 (88.0)	180 (90.5)	85 (83.3)	0.072
	Having problems	36 (12.0)	19 (9.5)	17 (16.7)	
**Usual activities**	No problems	260 (86.4)	179 (89.9)	81 (79.4)	**0.012**
	Having problems	41 (13.6)	20 (10.1)	21 (20.6)	
**Pain/discomfort**	No problems	132 (43.9)	105 (52.8)	27 (26.5)	**<0.001**
	Having problems	169 (56.1)	94 (47.2)	75 (73.5)	
**Anxiety/depression**	No problems	182 (60.5)	136 (68.3)	46 (45.1)	**<0.001**
	Having problems	119 (39.5)	63 (31.7)	56 (54.9)	
**Utility score** (mean±SD)		0.86±0.17	0.89±0.13	0.79±0.21	**<0.001**
**EQ-VAS score** (mean±SD)		69.12±12.60	71.13±11.78	65.15±13.20	**<0.001**
**DASS-10 score** (mean±SD)		5.04±6.38	4.82±6.34	5.47±6.47	0.245
EQ-VAS: EQ visual analogue scale, DASS-10: Depression Anxiety Stress Scale, SD: standard deviation.					

Fig 1. The proportion of patient responses by the extent of problems reported in each EQ-5D-5L domain among non-metastatic and metastatic HER2-positive breast cancer patients

Factors associated with the health-state utility of patients with HER2-positive breast cancer

The average utility score of patients aged 60 or older was 0.80±0.19, significantly lower than that of other age groups (p=0.001). This figure for illiterate patients and those only graduating from a primary school (0.69±0.25) was also significantly lower than that of patients with higher education levels (p<0.001). With regard to patients' occupations, the average utility score of employees and business people was the highest

(0.93±0.10), while that of houseworkers was the lowest (0.76±0.18) (p<0.001). Among income groups, patients with more than three million Vietnam dongs per month had the highest average utility score (0.90±0.11, p<0.001). Regarding clinical characteristics, lower average utility scores were witnessed among menopausal women (0.84±0.17, p=0.001) and patients receiving chemotherapy in combination with HER2-targeted therapy (0.80±0.23, p=0.026) (Table 4).

Table 4. Breast cancer patients' average utility scores by their demographic and clinical characteristics

Independent variables		Mean±SD	Min-Max	Median (25th-75th)	p-value
**Demographic characteristics**					
**Age**	<40	0.88±0.20	0.03-1.00	0.95 (0.85-1.00)	0.001
	40-49	0.89±0.12	0.56-1.00	0.92 (0.84-1.00)	
	50-59	0.85±0.17	0.03-1.00	0.88 (0.80-1.00)	
	≥60	0.80±0.19	0.03-1.00	0.85 (0.74-0.92)	
**Residence**	Urban	0.86±0.18	0.03-1.00	0.92 (0.78-1.00)	0.302
	Rural	0.85±0.16	0.03-1.00	0.89 (0.80-1.00)	
**Education level**	Primary school or lower	0.69±0.25	0.03-0.94	0.74 (0.58-0.87)	**<0.001**
	Secondary school	0.84±0.16	0.07-1.00	0.85 (0.78-0.93)	
	High school or college	0.86±0.17	0.03-1.00	0.92 (0.80-1.00)	
	University or higher	0.94±0.10	0.46-1.00	1.00 (0.90-1.00)	
**Job (occupation)**	Employment or business	0.93±0.10	0.46-1.00	0.98 (0.89-1.00)	**<0.001**
	Agriculture	0.85±0.15	0.18-1.00	0.85 (0.78-0.94)	
	Housework	0.76±0.18	0.37-1.00	0.85 (0.65-0.88)	
	Freelancers	0.81±0.22	0.03-1.00	0.85 (0.74-1.00)	
	Retired	0.87±0.16	0.03-1.00	0.92 (0.84-1.00)	

Independent variables		Mean±SD	Min-Max	Median (25th-75th)	p-value
**Marital status**	Unmarried or divorced	0.81±0.16	0.37-1.00	0.85 (0.74-0.94)	0.104
	Married	0.86±0.17	0.03-1.00	0.92 (0.80-1.00)	
**Giving birth**	No	0.91±0.15	0.73-1.00	1.00 (0.73-1.00)	0.498
	Yes	0.86±0.17	0.03-1.00	0.89 (0.80-1.00)	
**Patient monthly income (mVND)***	No income	0.81±0.18	0.07-1.00	0.85 (0.73-0.92)	**<0.001**
	≤ 2	0.80±0.18	0.18-1.00	0.81 (0.74-0.92)	
	> 2 to 3	0.79±0.25	0.03-1.00	0.85 (0.75-0.94)	
	> 3	0.90±0.11	0.56-1.00	0.92 (0.85-1.00)	
**Clinical characteristics**					
**Comorbidity**	Yes	0.87±0.17	0.07-1.00	0.92 (0.85-1.00)	0.338
	No	0.85±0.17	0.03-1.00	0.89 (0.78-1.00)	
**Menopause**	Yes	0.84±0.17	0.03-1.00	0.85 (0.78-0.94)	**0.001**
	No	0.88±0.17	0.03-1.00	0.92 (0.85-1.00)	
**Hormone receptor**	Positive	0.87±0.16	0.03-1.00	0.92 (0.83-1.00)	0.077
	Negative	0.84±0.18	0.03-1.00	0.85 (0.78-1.00)	
**Treatment regimen**	Chemotherapy only	0.87±0.11	0.36-1.00	0.85 (0.81-0.96)	**0.026**
	Chemotherapy + targeted therapy	0.80±0.23	0.03-1.00	0.85 (0.72-1.00)	
	Targeted therapy with or without hormone therapy	0.88±0.16	0.03-1.00	0.92 (0.85-1.00)	

*1 mVND (million Vietnam dongs) = 41.27 US dollars. SD: standard deviation.

As per the results of the Tobit regression model, the health-state utility of patients with HER-2 positive breast cancer was significantly associated with their age, education level, monthly income, stage of cancer (non-metastatic or metastatic), EQ-VAS score, and DASS-10 score. The higher the patient's age and the DASS-10 score, the lower the utility scores (negative correlations). For each one-unit increase in patient's age (one year) and DASS-10 score (one score), their utility scores decreased by 0.005 (p=0.003) and 0.007 (p<0.001), respectively. In contrast, there was a positive correlation between the EQ-VAS scores and health-state utility (beta=0.007, p<0.001). Patients' utility scores also increased by 0.008 for each one-unit monthly income increase (one million Vietnam dongs) (p=0.027). In addition, higher utility scores were found among patients with an education level of university or higher (p=0.004) and non-metastatic breast cancer patients (p<0.001) (Table 5).

Table 5. Factors associated with the health-state utility of patients with HER2-positive breast cancer (Tobit regression analyses)

Independent variable		Univariate		Multivariate	
		coef.	p-value	a.coef.	p-value
**Age** (a numeric variable)		-0.006	**<0.001**	-0.005	**0.003**
**Education level** (ref: primary school or lower)	Secondary school	0.174	**0.003**	0.064	0.156
	High school or college	0.224	**<0.001**	0.090	0.054
	University or higher	0.364	**<0.001**	0.174	**0.004**
**Job (occupation)** (ref: Housework)	Employment or business	0.247	**0.001**	-0.059	0.258
	Agriculture	0.106	**0.039**	0.062	0.127
	Freelancers	0.076	0.145	-0.048	0.257
	Retired	0.135	**0.019**	-0.003	0.987
**Patient monthly income (mVND)*** (a numeric variable)		0.019	**<0.001**	0.008	**0.027**
**Menopause** (ref: No)	Yes	-0.075	**0.007**	0.033	0.240
**Metastasis** (ref: No)	Yes	-0.149	**<0.001**	-0.084	**<0.001**

Independent variable		Univariate		Multivariate	
		**coef.**	**p-value**	**a.coef.**	**p-value**
**Treatment regimen** (ref: HER2-targeted therapy with or without hormone therapy)	Chemotherapy only	-0.036	0.252	0.034	0.185
	Chemotherapy + HER2-targeted therapy	-0.112	**0.001**	-0.039	0.143
**EQ-VAS score** (a numeric variable)		0.009	**<0.001**	0.007	**<0.001**
**DASS-10 score** (a numeric variable)		-0.012	**<0.001**	-0.007	**<0.001**

*1 mVND (million Vietnam dongs) = 41.27 US dollars. Ref: reference, coef.: coefficient, a.coef.: adjusted coefficient, EQ-VAS: EQ visual analogue scale, DASS-10: Depression Anxiety Stress Scale.

The multicollinearity was checked using the Variance Inflation Factor (VIF) for each independent variable. In the multivariate model, all VIF values were lower than 2.5.

Discussion

This study was conducted to measure HER2-positive breast cancer patients' health-state utility and associated factors in Vietnam using the EQ-5D-5L instrument. Our findings showed that the average utility score of 301 patients with HER2-positive breast cancer was 0.86±0.17. This figure among non-metastatic breast cancer patients (0.89±0.13) was significantly higher than that of the metastatic cancer patient group (0.79±0.21). The utility scores were positively correlated with patients' monthly income and EQ-VAS score but negatively correlated with their age and level of distress (DASS-10 score). In addition, education level was another factor associated with the health-state utility of these patients.

Patients' health profile across five health dimensions of EQ 5D-5L and DASS-10

Pain/discomfort and anxiety/depression were two common health issues that many Vietnamese patients with HER2-positive breast cancer suffered from (56.1% and 39.5%, respectively). The number of patients with severe or extreme problems with these two dimensions was also the highest. By contrast, not many patients had problems involving self-care (12.0%). These results were in line with the findings of numerous previous studies in Brazil, China, Ethiopia, Korea, and Singapore [14, 24-29]. However, anxiety/depression was the most common health issue among breast cancer patients in India and Indonesia, followed by pain/discomfort [30, 31].

In the United Kingdom, usual activities and pain/discomfort were two common issues of HER2-positive breast cancer patients [32]. The differences in the findings of studies can be explained by the differences in studying time, location, stage of cancer, and the sample. Besides, in spite of the high number of Vietnamese breast cancer patients having problems with anxiety/depression, the severity of this health dimension was relatively mild (according to the DASS-10 scores). Nonetheless, both physical and mental health should be paid attention to when taking care of breast cancer patients.

For all five dimensions, the percentage of patients having problems in the metastatic group was higher than that in the non-metastatic group. However, the differences were only significant in three dimensions, including usual activities, pain/discomfort, and anxiety/depression. Furthermore, it is perfectly understandable that the severity of these health dimensions among the former was greater than that of the latter. Two studies in Brazil and China reported similar results [26, 29]. In the United Kingdom, the differences among patient groups were statistically significant in morbidity, self-care, and usual activities. The other two dimensions (including pain/discomfort and anxiety/depression) did not find any significant differences [32]. The findings from a study in the United States also showed that the spiritual and emotional quality of life outcomes among advanced breast cancer women were not significantly different as per metastatic status [33].

Patients' EQ-VAS scores

The average EQ-VAS score of Vietnamese patients with HER2-positive breast cancer was 69.12, in line with the result of a study in Ethiopia (69.9) [28]. Our result was slightly higher than the result of a previous study in Vietnam (64.9) [14] but far lower than the result of a study among Chinese patients (80.0) [25]. This difference may spring from the differences between the sample, location, studying time, and treatment therapies. Regarding the stages of cancer, in our research, there was a significant difference in the EQ-VAS scores between patients with metastatic breast cancer (65.20) and those with non-metastatic breast cancer (71.13). Our findings were similar to the results of a study in the United Kingdom (patients receiving treatment for metastatic breast cancer: 65.82 and patients currently undergoing treatment for early breast cancer: 72.74) [32]. In comparison with non-metastatic breast cancer patients, the higher EQ-VAS scores of patients with metastatic breast cancer were also reported in Brazil, China, India, and Indonesia [25, 26, 29-31].

Health-state utility and associated factors

Of all 301 Vietnamese patients with HER2-positive breast cancer, their average utility score was 0.86, consistent with the results of studies in China, Ethiopia, and India [25, 28, 30, 34] but higher than the findings of

a study in Singapore [27]. In comparison with the utility score of breast cancer patients from a previous study in Vietnam in 2019, our result was higher (0.74 and 0.86, respectively) [14]. Used treatment regimens can be the rationale behind the difference between the two studies. In this study, we only recruited patients receiving systemic treatment. Meanwhile, in the study in 2019, breast cancer patients could receive various kinds of treatment therapies, such as lumpectomy, mastectomy, and breast reconstruction surgery.

The stage of cancer/metastatic status is a crucial factor affecting treatment targets and regimens for breast cancer patients. The average utility scores of metastatic and non-metastatic breast cancer patients in Vietnam were 0.79 and 0.89, higher than those of HER2-positive breast cancer patients in the United Kingdom (0.70 and 0.81), respectively [32]. For both metastatic and non-metastatic cancer groups, there were also differences in the utility scores of patients among countries (such as the Netherlands and Sweden [35], Korea [36], China [25, 37], Indonesia [31]), but the gaps were not huge. In addition, previous studies showed that the health-related quality of life and health-state utility of patients with metastatic breast cancer were significantly lower than those of patients with early-stage and non-metastatic breast cancer [38, 39]. As a result, it is necessary to have screening programs to detect and diagnose breast cancer at an early stage and have suitable healthcare programs prioritized for late-stage cancer patients.

In this study, three demographic characteristics of Vietnamese patients associated with their health-state utility were age, education level, and monthly income. In Asia, breast cancer patients receiving chemotherapy, having comorbidities, less social support, and more unmet needs had poorer health-related quality of life [38]. In low- and middle-income countries in Asia, breast cancer patients' health-related quality of life was associated with their age, marital status, education level, income, stage of the tumor, method, treatment duration, and lifestyle [40]. Similar to our results, the poor health-related quality of life and health-state utility of breast cancer patients with old age, low education level, and low income per month were also found among patients in other countries, such as China, Korea, and India [14, 26, 30, 36, 41, 42]. However, in several studies in China and Japan, younger patients had lower health-related quality of life by reason of being under a lot of pressure and stress [43-45]. With regard to patients' monthly income and education level, our findings were in line with the results of a systematic review: those with higher income and education levels had higher health-related quality of life [40]. Patients with low income and low education levels may be unable to afford the treatment costs and not comprehend the crucial role of treatment adherence, thereby lowering the treatment's effectiveness and efficacy. For patients living on the breadline, health insurance can play an essential role in their treatment process. Sadly,

in Vietnam, health insurance does not cover the treatment costs of nearly all HER2-targeted drugs, except for trastuzumab (with a reimbursement rate of 60%). To improve HER2-positive breast cancer patients' health outcomes and health-related quality of life, the government, policymakers, and other relevant parties (especially in low- and middle-income countries) should have practical policies and solutions to support patients in their long-term treatment period.

Besides the four factors above, the health-state utility of HER2-positive breast cancer patients in Vietnam was also associated with their EQ-VAS score and level of distress (DASS-10 score). Among patients with metastatic breast cancer in Germany, their EQ-VAS scores were significantly associated with single EQ-5D-5L items and the total score [46]. Furthermore, previous studies demonstrated that breast cancer patients suffering from depression, anxiety, and stress also had lower health-related quality of life [12, 47, 48]. These burdens highlighted the essential roles of physical and mental health care for breast cancer patients, especially for the metastatic cancer group. Improving patients' physical and mental health is of paramount importance [49].

Strengths and limitations

This is the first study conducted in Vietnam to measure the health-state utility of 301 HER2-positive breast cancer patients in two health facilities. The reliability and validity of the instruments were demonstrated. Information about patients' health profiles, the division of patients into metastatic and non-metastatic cancer groups to analyze data, and factors associated with their health-related quality of life can contribute to developing healthcare programs and supporting their treatment process. In addition, the utility scores computed from the EQ-5D-5L instrument for Vietnamese patients receiving systemic treatment can be used in the health technology assessment of HER2-target drugs, especially in case of the heterogeneity in the utility scores of breast cancer patients among countries around the world. Besides the strengths above, our research has several limitations. First, since patients with HER2-positive breast cancer only account for approximately 15-20% of breast cancer patients, our sample size was not high. Second, collecting data via patient interviews can bring several biases, such as recall bias. Missing values from questions about the monthly income of patients and their families decreased the sample size in univariate and multivariate regression analyses. Furthermore, by virtue of being a cross-sectional study, our results only reflect patients' information at a point in time, and the causal relationship between the health-state utility and independent factors cannot be determined.

Conclusions

Many patients with HER2-positive breast cancer had problems involving pain/discomfort and anxiety/depression, especially among metastatic breast cancer patients. Patients' utility scores were relatively high, and their health issues involving depression, anxiety, and stress were relatively mild. Factors associated with patients' health-state utility included their age, education level, monthly income, metastatic status, EQ-VAS score, and level of distress. Both physical and mental health should be paid attention to when taking care of breast cancer patients. Our findings provide important information that can be used in the health technology assessment of HER2-directed therapy and make a contribution to supporting patients during their long treatment period.

Acknowledgments

We would like to thank the Board of Directors of the Vietnam National Cancer Hospital and the Hue Central Hospital, doctors, nurses, and patients for their participation and support during the data collection process.

Funding

This research received no specific grant from any funding agency in the public, commercial, or not-for-profit sectors.

Conflict of interest

The authors declare that they have no competing interests.

Author contributions

TNNL: Conceptualization, Methodology, Investigation, Data curation, Formal analysis, Visualization, Writing – Original Draft Preparation, Writing – Review & Editing. **DXD**: Conceptualization, Methodology, Formal analysis, Visualization, Supervision, Project administration, Validation, Writing – Original Draft Preparation, Writing – Review & Editing. **TXT**: Conceptualization, Investigation, Formal analysis, Writing – Review & Editing. **TBT**: Conceptualization, Methodology, Formal analysis, Writing – Review & Editing. **HTT**: Conceptualization, Methodology, Supervision, Project administration, Writing – Review & Editing. **KHTP**: Conceptualization, Methodology,

Supervision, Project administration, Writing – Review & Editing. **HTTN**: Conceptualization, Methodology, Investigation, Data curation, Supervision, Project administration, Writing – Review & Editing.

References

1. Sung H, Ferlay J, Siegel RL, Laversanne M, Soerjomataram I, Jemal A, et al. Global Cancer Statistics 2020: GLOBOCAN Estimates of Incidence and Mortality Worldwide for 36 Cancers in 185 Countries. CA Cancer J Clin. 2021;71(3):209-249. doi: 10.3322/caac.21660.

2. International Agency for Research on Cancer, World Health Organization. Viet Nam. 2021 Mar [Cited 2023 Dec 13]. Available from: https://gco.iarc.fr/today/data/factsheets/populations/704-viet-nam-fact-sheets.pdf

3. World Health Organization. Breast cancer. 2023 Jul 12 [Cited 2023 Dec 13]. Available from: Available from: https://www.who.int/news-room/fact-sheets/detail/breast-cancer

4. Klapper LN, Glathe S, Vaisman N, Hynes NE, Andrews GC, Sela M, et al. The ErbB-2/HER2 oncoprotein of human carcinomas may function solely as a shared coreceptor for multiple stroma-derived growth factors. Proc Natl Acad Sci U S A. 1999;96(9):4995-5000. doi: 10.1073/pnas.96.9.4995.

5. Hicks DG, Kulkarni S. HER2+ breast cancer: review of biologic relevance and optimal use of diagnostic tools. Am J Clin Pathol. 2008;129(2):263-73. doi: 10.1309/99AE032R9FM8WND1.

6. Krishnamurti U, Silverman JF. HER2 in breast cancer: a review and update. Adv Anat Pathol. 2014;21(2):100-7. doi: 10.1097/PAP.0000000000000015.

7. Swain SM, Shastry M, Hamilton E. Targeting HER2-positive breast cancer: advances and future directions. Nat Rev Drug Discov. 2023;22(2):101-126. doi: 10.1038/s41573-022-00579-0.

8. Loibl S, Gianni L. HER2-positive breast cancer. Lancet. 2017;389(10087):2415-2429. doi: 10.1016/S0140-6736(16)32417-5.

9. Ahmed S, Sami A, Xiang J. HER2-directed therapy: current treatment options for HER2-positive breast cancer. Breast Cancer. 2015;22(2):101-16. doi: 10.1007/s12282-015-0587-x.

10. Maximiano S, Magalhães P, Guerreiro MP, Morgado M. Trastuzumab in the Treatment of Breast Cancer. BioDrugs. 2016;30(2):75-86. doi: 10.1007/s40259-016-0162-9.

11. Yu Q, Zhu Z, Liu Y, Zhang J, Li K. Efficacy and Safety of HER2-Targeted Agents for Breast Cancer with HER2-Overexpression: A Network Meta-Analysis. PLoS One. 2015;10(5):e0127404. doi: 10.1371/journal.pone.0127404.

12. Tran TH, Trinh NL, Hoang Y, Nguyen TL, Vu TT. Health-Related Quality of Life Among Vietnamese Breast Cancer Women. Cancer Control. 2019;26(1):1073274819862787. doi: 10.1177/1073274819862787.

13. Tran TN, Pham NT, Phan Thi DQ, Nguyen HS. Quality of Life and Its Contributing Factors among Breast Cancer Patients after Intensive Treatment. Ann Cancer Res Ther. 2020;28(2):102–6. doi:10.4993/acrt.28.102.

14. Ngan TT, Mai VQ, Van Minh H, Donnelly M, O'Neill C. Health-related quality of life among breast cancer patients compared to cancer survivors and age-matched women in the general population in Vietnam. Qual Life Res. 2022;31(3):777-787. doi: 10.1007/s11136-021-02997-w.

15. Guo YQ, Ju QM, You M, Liu Y, Yusuf A, Soon LK. Depression, anxiety and stress among metastatic breast cancer patients on chemotherapy in China. BMC Nurs. 2023;22(1):33. doi: 10.1186/s12912-023-01184-1.

16. Adeyemi OJ, Gill TL, Paul R, Huber LB. Evaluating the association of self-reported psychological distress and self-rated health on survival times among women with breast cancer in the U.S. PLoS One. 2021;16(12):e0260481. doi: 10.1371/journal.pone.0260481.

17. Javan Biparva A, Raoofi S, Rafiei S, Masoumi M, Doustmehraban M, Bagheribayati F, et al. Global depression in breast cancer patients: Systematic review and meta-analysis. PLoS One. 2023;18(7):e0287372. doi: 10.1371/journal.pone.0287372.

18. Kim Halford W, Frost ADJ. Depression Anxiety Stress Scale-10: A Brief Measure for Routine Psychotherapy Outcome and Progress Assessment. Behav Change. 2021;38(4):221-234. doi:10.1017/bec.2021.12.

19. NovoPsych. Depression Anxiety Stress Scale (DASS-10). [Cited 2023 Dec 13]. Available from: https://novopsych.com.au/assessments/depression/depression-anxiety-stress-scale-dass-10/

20. Kennedy-Martin M, Slaap B, Herdman M, van Reenen M, Kennedy-Martin T, Greiner W, et al. Which multi-attribute utility instruments are recommended for use in cost-utility analysis? A review of national health technology assessment (HTA) guidelines. Eur J Health Econ. 2020;21(8):1245-1257. doi: 10.1007/s10198-020-01195-8.

21. EuroQol Research Foundation. EQ-5D-5L. 2023 Dec 13 [Cited 2023 Dec 13]. Available from: https://euroqol.org/eq-5d-instruments/eq-5d-5l-about/

22. Mai VQ, Sun S, Minh HV, Luo N, Giang KB, Lindholm L, et al. An EQ-5D-5L Value Set for Vietnam. Qual Life Res. 2020;29(7):1923-1933. doi: 10.1007/s11136-020-02469-7.

23. Tran BT, Pham NH, Nguyen TX, Choi KS, Sohn DK, Kim SY, et al. Measurement of Health-Related Quality of Life Among Colorectal Cancer Patients Using the Vietnamese Value Set of the EQ-5D-5L. Patient Prefer Adherence. 2020 Dec 4;14:2427-2437. doi: 10.2147/PPA.S281500.

24. Kim SH, Jo MW, Lee JW, Lee HJ, Kim JK. Validity and reliability of EQ-5D-3L for breast cancer patients in Korea. Health Qual Life Outcomes. 2015;13:203. doi: 10.1186/s12955-015-0399-x.

25. Yang Q, Yu X, Zhang W. Health variations among breast-cancer patients from different disease states: evidence from China. BMC Health Serv Res. 2020;20(1):1033. doi: 10.1186/s12913-020-05872-5.

26. Wang L, Shi JF, Zhu J, Huang HY, Bai YN, Liu GX, et al. Health-related quality of life and utility scores of patients with breast neoplasms in China: A multicenter cross-sectional survey. Breast. 2018;39:53-62. doi: 10.1016/j.breast.2018.03.004.

27. Lee CF, Ng R, Luo N, Wong NS, Yap YS, Lo SK, et al. The English and Chinese versions of the five-level EuroQoL Group's five-dimension questionnaire (EQ-5D) were valid and reliable and provided comparable scores in Asian breast cancer patients. Support Care Cancer. 2013;21(1):201-9. doi: 10.1007/s00520-012-1512-x.

28. Sibhat SG, Fenta TG, Sander B, Gebretekle GB. Health-related quality of life and its predictors among patients with breast cancer at Tikur Anbessa Specialized Hospital, Addis Ababa, Ethiopia. Health Qual Life Outcomes. 2019;17(1):165. doi: 10.1186/s12955-019-1239-1.

29. Monteiro MR, Nunes NCC, Crespo J, Abrahão ABK, Buscacio G, Lerner LCC, et al. Patient-centered Outcomes in Breast Cancer: Description of EQ-5D-5L and EORTC-QLQ-BR23 Measurements in Real-world Data and Their Association With Survival. Clin Oncol (R Coll Radiol). 2022;34(9):608-616. doi: 10.1016/j.clon.2022.05.015.

30. Wadasadawala T, Mohanty SK, Sen S, Khan PK, Pimple S, Mane JV, et al. Health-Related Quality of Life (HRQoL) Using EQ-5D-5L: Value Set Derived for Indian Breast Cancer Cohort. Asian Pac J Cancer Prev. 2023;24(4):1199-1207. doi: 10.31557/APJCP.2023.24.4.1199.

31. Rukminingsih F, Andayani T, Rahmawati F, Widayati K. Health-related quality of life in early breast cancer patients with hormone responsive. Int J Pharm Pharm Sci. 2018;10:47. 10.22159/ijpps.2018v10i12.29648.

32. Verrill M, Wardley AM, Retzler J, Smith AB, Bottomley C, Ní Dhochartaigh S, et al. Health-related quality of life and work productivity in UK patients with HER2-positive breast cancer: a cross-sectional study evaluating the relationships between disease and treatment stage. Health Qual Life Outcomes. 2020;18(1):353. doi: 10.1186/s12955-020-01603-w.

33. Wyatt G, Sikorskii A, Tamkus D, You M. Quality of life among advanced breast cancer patients with and without distant metastasis. Eur J Cancer Care (Engl). 2013;22(2):272-80. doi: 10.1111/ecc.12028.

34. Liu L, Li S, Wang M, Chen G. Comparison of EQ-5D-5L health state utilities using four country-specific tariffs on a breast cancer patient sample in mainland China. Patient Prefer Adherence. 2017;11:1049-1056. doi: 10.2147/PPA.S138028.

35. Frederix GW, Quadri N, Hövels AM, van de Wetering FT, Tamminga H, Schellens JH, et al. Utility and work productivity data for economic evaluation of breast cancer therapies in the Netherlands and Sweden. Clin Ther. 2013;35(4):e1-7. doi: 10.1016/j.clinthera.2013.03.009.

36. Park M, Yu SY, Jeon HL, Song I. Factors Affecting Health-Related Quality of Life in Patients With Metastatic Breast Cancer. J Breast Cancer. 2023;26(5):436-445. doi: 10.4048/jbc.2023.26.e29.

37. Rautenberg T, Hodgkinson B, Zerwes U, Downes M. Meta-analysis of health state utility values measured by EuroQol 5-dimensions (EQ5D) questionnaire in Chinese women with breast cancer. BMC Cancer. 2022;22(1):52. doi: 10.1186/s12885-021-09140-5.

38. Ho PJ, Gernaat SAM, Hartman M, Verkooijen HM. Health-related quality of life in Asian patients with breast cancer: a systematic review. BMJ Open. 2018;8(4):e020512. doi: 10.1136/bmjopen-2017-020512.

39. Gonzalez L, Bardach A, Palacios A, Peckaitis C, Ciapponi A, Pichón-Riviere A, et al. Health-Related Quality of Life in Patients with Breast Cancer in Latin America and the Caribbean: A Systematic Review and Meta-Analysis. Oncologist. 2021;26(5):e794-e806. doi: 10.1002/onco.13709.

40. Ngo NTN, Nguyen HT, Nguyen PTL, Vo TTT, Phung TL, Pham AG, et al. Health-related quality of life in breast cancer patients in low-and-middle-income countries in Asia: a systematic review. Front Glob Womens Health. 2023;4:1180383. doi: 10.3389/fgwh.2023.1180383.

41. Yan B, Yang LM, Hao LP, Yang C, Quan L, Wang LH, et al. Determinants of Quality of Life for Breast Cancer Patients in Shanghai, China. PLoS One. 2016;11(4):e0153714. doi: 10.1371/journal.pone.0153714.

42. Imran M, Al-Wassia R, Alkhayyat SS, Baig M, Al-Saati BA. Assessment of quality of life (QoL) in breast cancer patients by using EORTC QLQ-C30 and BR-23 questionnaires: A tertiary care center survey in the western region of Saudi Arabia. PLoS One. 2019;14(7):e0219093. doi: 10.1371/journal.pone.0219093.

43. So WK, Choi KC, Chan CW, Chair SY. Age-related differences in the quality of life of Chinese women undergoing adjuvant therapy for breast cancer. Res Gerontol Nurs. 2011;4(1):19-26. doi: 10.3928/19404921-20101201-01.

44. Takada K, Kashiwagi S, Asano Y, Goto W, Morisaki T, Takahashi K, et al. Significance of age-associated quality of life in patients with stage IV breast cancer who underwent endocrine therapy in Japan. Oncol Lett. 2020;20(5):180. doi: 10.3892/ol.2020.12041.

45. Javan Biparva A, Raoofi S, Rafiei S, Pashazadeh Kan F, Kazerooni M, Bagheribayati F, et al. Global quality of life in breast cancer: systematic review and meta-analysis. BMJ Support Palliat Care. 2022:bmjspcare-2022-003642. doi: 10.1136/bmjspcare-2022-003642.

46. Brusniak K, Feisst M, Sebesteny L, Hartkopf A, Graf J, Engler T, et al. Measuring the Time to Deterioration for Health-Related Quality of Life in Patients With Metastatic Breast Cancer Using a Web-Based Monitoring Application: Longitudinal Cohort Study. JMIR Cancer. 2021;7(4):e25776. doi: 10.2196/25776.

47. Ng CG, Mohamed S, See MH, Harun F, Dahlui M, Sulaiman AH, et al. Anxiety, depression, perceived social support and quality of life in Malaysian breast cancer patients: a 1-year prospective study. Health Qual Life Outcomes. 2015;13:205. doi: 10.1186/s12955-015-0401-7.

48. An Y, Fu G, Yuan G. Quality of Life in Patients With Breast Cancer: The Influence of Family Caregiver's Burden and the Mediation of Patient's Anxiety and Depression. J Nerv Ment Dis. 2019;207(11):921-926. doi: 10.1097/NMD.0000000000001040.

49. Bates-Fraser LC, Riley S, Stopforth C, Moertl K, Edgar K, Stoner L, et al. Home-based exercise improves quality of life in breast and prostate cancer survivors: A meta-analysis. PLoS One. 2023;18(4):e0284427. doi: 10.1371/journal.pone.0284427.

Figure 1. The proportion of patient responses by the extent of problems reported in each EQ-5D-5L domain among non-metastatic and metastatic HER2-positive breast

Click here to access/download;Figure;Fig 1.tif

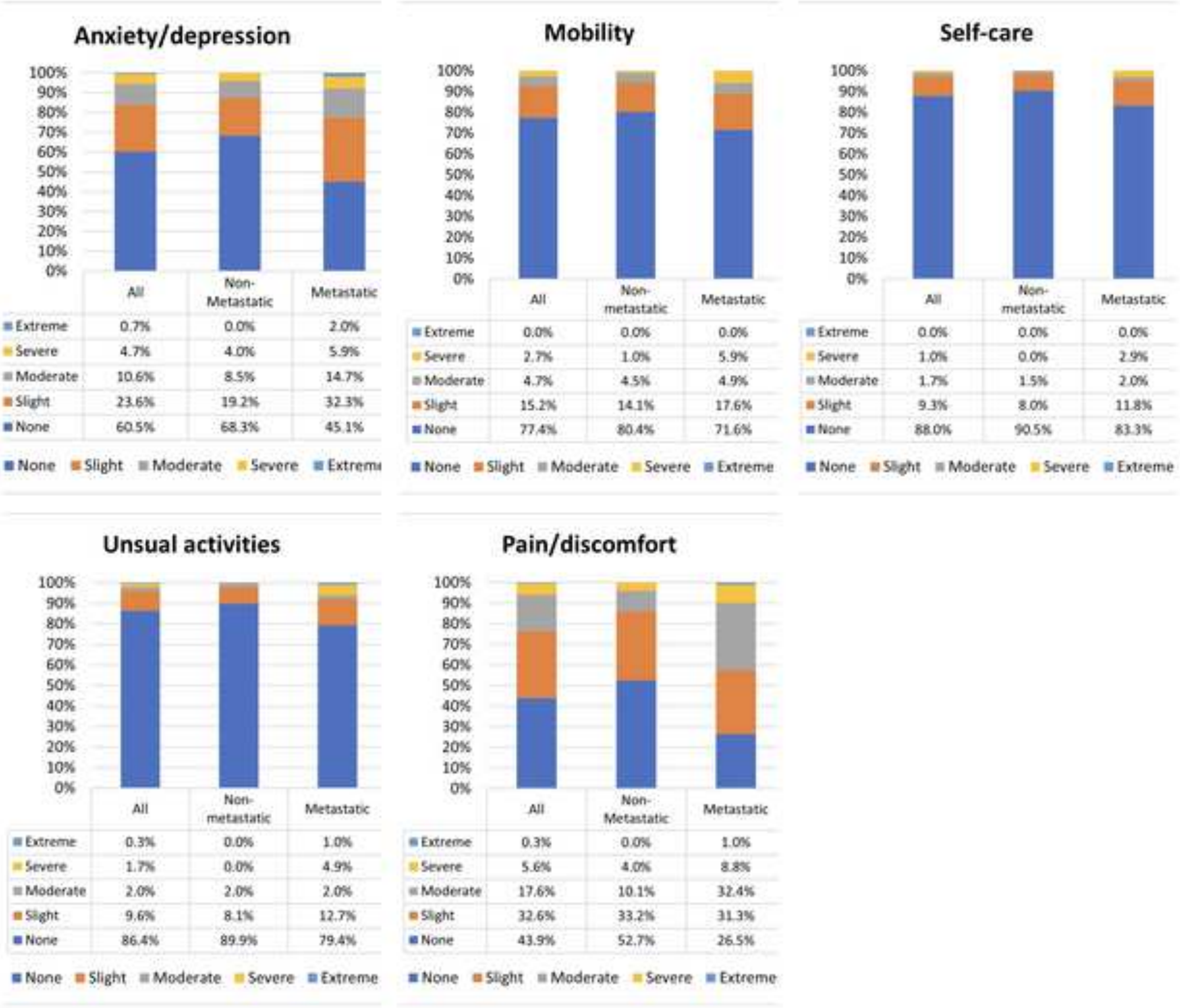

The original approval document (Vietnamese) and an English translation

Click here to access/download

Other

Ethics.pdf
