# Supplementary material for: Health-state utility of patients with HER2-positive breast cancer in Vietnam: A multicenter cross-sectional study
Source: PLoS One. 2024 May 14;19(5):e0303011. doi: 10.1371/journal.pone.0303011 (PMC11093329; doi:10.1371/journal.pone.0303011)
Supplement: Attachment — Submitted filename: Response to Reviewers.pdf [file pone.0303011.s003.pdf]

First and foremost, we want to express our deep gratitude to the editors and reviewers who gave us valuable and practical advice. We carefully read your comments and revised our manuscript. Our responses are written in blue color.

Thank you so much for your assistance.

....

REVIEWER REPORTS

**Reviewer #1:** The findings of this study based on breast cancer patients in Viet Nam can add value as the literature suggests that there are differences in health-related quality of life of the patients across countries. However, some revisions and clarifications would benefit the paper. Some of suggestions are listed as follows.<br><br>Abstract-Objective<br>Please add a little background for conducting this study.<br><br>We added some information about reasons for conducting our study in the Abstract.
Introduction<br>Lines 72-73, " The number of studies … is negligible." This statement seems to indicate there are existing studies. If that is the case, please cite some references and explain why there is a need for this current study based on the shortcomings of the previous studies.<br><br>We cited two previous studies about HER2-positive breast cancer patients in the UK and Brazil.
Methods<br>Lines 95-96, do standard hospital beds differ from inpatient beds? Please explain the difference as succinctly as possible. Also the authors may want to leave out the detail.<br><br>We revised this issue. They are similar. We used a general term (hospital bed) for these data.

Lines 109-111, please rewrite the sentence, which has grammatical and punctuation errors.<br><br>We revised this sentence.
Acronyms come after, not before, their full terms. However, the authors switched the order. For example, it should be the Depression Anxiety Stress Scale (DASS-10), not the other way around.<br><br>We revised this issue.
In lines 121-122, do the authors mean to include both personal and household income? This seems unnecessary and problematic. However, the results section seems to indicate that only the personal income was entered into the regression model. Please clarify.<br><br>We removed the variable "household monthly income" in the whole manuscript. We collected data on both patient and household monthly income. Sadly, there were many missing values about household monthly income (51 out of 301 participants). Numerous missing values can lower the quality of regression models. As a consequence, we removed this variable.
Line 135, in "… use in national health technology guidelines," please be more specific which nation recommends using EQ-5D-5L. This statement can be misinterpreted as Vietnamese government's guidelines.<br><br>We revised this issue.<br>In the article cited, "Thirty-four pharmacoeconomic guidelines were included for review. Multi-attribute utility instruments named for use in cost-utility analysis: EQ-5D (n = 29 guidelines), the SF-6D (n = 11), HUI (n = 10), QWB (n = 3), AQoL (n = 2), CHU9D (n = 1). EQ-5D was a preferred multi-attribute utility instrument in 15 guidelines."

Kennedy-Martin M, Slaap B, Herdman M, van Reenen M, Kennedy-Martin T, Greiner W, et al. Which multi-attribute utility instruments are recommended for use in cost-utility analysis? A review of national health technology assessment (HTA) guidelines. Eur J Health Econ. 2020;21(8):1245-1257. doi: 10.1007/s10198-020-01195-8.
The top line of Table 2 is mislabeled. It should not be "demographic characteristics." In Table 3 and in line 250, there should be mobility instead of morbidity.<br>In general, this manuscript would benefit from thorough spell and grammatical checks.<br><br>We corrected these mistakes.<br>We endeavoured to check linguistic issues. Thank you for this helpful comment.
Discussion<br>Lines 245-253, please try to explain potential reasons for similarities between Viet Nam and Brazil/China and for differences between Viet Nam and the U.K/U.S.<br><br>We added some reasons for these points.
Lines 270-272 seems to hint a rationale for conducting this present study which relied on patients on pharmacological treatment only whereas the 2019 study derived utility scores from Vietnamese patients who underwent surgery as well. This point could have been used to reinforce the need for the current study in the introduction section.<br><br>Thank you for this helpful comment.

If there is anything inappropriate or inadequate, please let us know. Thank you so much for your valuable comments.

Best regards.

**Reviewer #2:** This is a sound piece of scientific research highlighting population characteristics of HER2 positive breast cancer patients in Vietnam. The authors do not make their data available due to potentially identifiable information, which is reasonable in the context of this manuscript.<br><br>The data file is included in the Supporting Information files. We removed the names of patients and shared de-identified or anonymized data to make a small contribution to the scientific integrity.
Abstract: Conclusions are too broad, please revise this to directly reflect the study results<br><br>We revised the Conclusion in the Abstract.
Lines 47 – 49: Insert reference<br><br>We revised this issue.
Lines 47 – 53: newer data?<br><br>Data in 2022 were added.
Line 111 – 114: time during which of data collection occurred for individual patient should be reported, (pre-treatment or post-treatment).<br><br>We added some information about the time during which of data collection occurred.

If there is anything inappropriate or inadequate, please let us know. Thank you so much for your valuable comments.

Best regards.
