# Supplementary material for: Health-state utility of patients with HER2-positive breast cancer in Vietnam: A multicenter cross-sectional study
Source: PLoS One. 2024 May 14;19(5):e0303011. doi: 10.1371/journal.pone.0303011 (PMC11093329; doi:10.1371/journal.pone.0303011)
Supplement: Attachment — Submitted filename: Response to Reviewers.docx [file pone.0303011.s004.docx]

First and foremost, we want to express our deep gratitude to the editors and reviewers who gave us valuable and practical advice. We carefully read your comments and revised our manuscript. Our responses are written in blue color.

Thank you so much for your assistance.

..........................................................................................................................

**REVIEWER REPORTS**

**Reviewer #1:** Congrats on successful revisions.

P in p values should be italicized

Check the spelling -> immunohistochemistry

We revised these issues.

**Reviewer #3:** The authors have done a reasonable job of responding to the earlier comments. However, this reviewer has one important comment i.e, lack of adjustment for time since diagnosis. The physical and mental health can vary substantially based on how long ago the patients were diagnosed with breast cancer (prior to their study enrollment and interview). It is recommended that the authors obtain this information and incorporate it into all their analysis.

Regarding the time since the first diagnosis, we did collect data on this variable last year. However, when we analyzed the data, the association between patients’ utility and their time since the first diagnosis was insignificant (p-value>0.05). Therefore, when we drafted the initial manuscript, we did not report this variable.

As per the comment of Reviewer 3, we added several results involving this variable to the revised manuscript. It is noted that although in Table 4, there was a significant difference among the utility scores of patient groups (this variable was divided into four groups – a categorical variable). However, in the univariate linear regression model, when it is a numeric variable, the p-value is higher than 0.05 (coef=-0.001, p=0.059).

As we can see from the results in Table 5, other variables, such as the stage of cancer/metastatic status, may have a stronger association with patients’ utility than the time since the first diagnosis of breast cancer. It is perfectly understandable. For example, the time since the first diagnosis of breast cancer of a patient is three months. However, this diagnosis is late and this person is a metastatic patient.

If there is anything inappropriate or inadequate, please let us know. Thank you so much for your valuable comments.

Best regards.
